# Sustainability criteria: their indicators, control, and monitoring (with examples from the biofuel sector)

**DOI:** 10.1186/s12302-014-0017-2

**Published:** 2014-07-23

**Authors:** Evgenia Pavlovskaia

**Affiliations:** Faculty of Law, Lund University, P.O. Box 207, Lund, 22100 Sweden

**Keywords:** Sustainability criteria, Indicators for sustainability criteria, Control of the fulfillment, Monitoring

## Abstract

**Background:**

The purpose of the article is to research and analyze the notion of sustainability criteria in their function of an emerging tool to promote and safeguard sustainable products and their sustainable production. The article addresses critical issues, which are important for deeper understanding of sustainability criteria and their practical use. In this, the article examines the existing definitions of sustainability criteria, explores what indicators for sustainability criteria are, researches the issue of costs for following sustainability criteria, and discusses what groups of actors can be responsible for setting and supporting sustainability criteria. The research is done from a legal perspective, which involves much attention on how sustainability criteria can efficiently be implemented and used in legal constructions. Examples from the biofuel sector, which is regulated through a variety of legal frameworks and voluntary sustainability standards with sustainability criteria, are provided.

**Results:**

The research results highlight that sustainability criteria is not a clearly defined concept. Their content should be linked to the understanding of what sustainable development and sustainability in each particular branch are. Purposes of sustainability criteria have to be explained and clarified so that it is easier to interpret and fulfill them. In some cases, sustainability criteria can set an upper limit to the use of natural resources and provide institutional guidance.

**Conclusions:**

It is desirable that sustainability criteria are applied at initial stages of an industry development. Control of how sustainability criteria are fulfilled and its quality are very important. Thoroughly elaborated regulations on control mechanisms and their components, such as monitoring, reporting, verification, and transparency, should be included into legal frameworks and voluntary sustainability standards. Different groups of actors at different levels can be responsible for setting and supporting the implementation of sustainability criteria. Among them are international institutions, states, their governments, independent bodies established by states, NGOs, producers, and users. Collaboration between these groups should be promoted.

## Background

The issue of developing sustainable products and their sustainable production throughout the entire product life cycle, addressing environmental, social, and economic sustainability, has become one of the main challenges in our time [1]. Sustainability requirements can be regulated with the help of different operational tools, e.g., sustainability criteria, sustainability standards, eco-labels, or their combinations. The purpose of the present article is to research and analyze the notion of sustainability criteria in their function of a tool to promote and safeguard sustainable products and their sustainable production.

More deeply, the article aims to explore the existing definitions of sustainability criteria, to investigate what indicators for sustainability criteria are, to address the issue of costs for following sustainability criteria, and to discuss what groups of actors can be responsible for setting and supporting sustainability criteria. Features special for the efficient use, implementation, and enforcement of sustainability criteria, such as control and monitoring of their fulfillment, are also highlighted and researched. Attention is paid to the fact that sustainability criteria cannot function as the only guarantee for the sustainable quality of a product and its sustainable production. They should be accompanied and completed by other tools and instruments.

The notion of sustainability criteria has been explored from various scientific perspectives in the previous research [2–6]. However, the achieved results have not been homogeneous or systematized. The nature of sustainability criteria for different, often incomparable sustainable products and production processes has been investigated. Terminology used in the previous research has not been homogeneous either.

In the article, the investigation is conducted from a legal perspective. This involves much consideration regarding how sustainability criteria can efficiently be implemented and used in legal constructions. It can be suggested that the analysis of the notion of sustainability criteria and methodology of their practical use need be developed further.

## Results and discussion

### Sustainability criteria: defining the concept

An international character of economy and importance of worldwide trade, which answers the demands of sustainable development, calls for the use of sustainability criteria for internationally traded products. The existence of sustainability criteria assures sustainability in the long perspective and secures investments. Another positive effect of introducing sustainability criteria is that products, which fulfill them, can later be linked to governmental subsidies [7]. For the best possible sustainability protection, it is desirable that sustainability criteria for a product and its production are applied at an earlier stage of an industry development.

The concepts of sustainable development and sustainability are the basis for understanding and defining sustainability criteria. Sustainable development was first described in 1987 by the Brundtland Commission as ‘development that meets the needs of the present without compromising the ability of future generations to meet their own needs’ [8,9]. Further interpretations of the concept ‘sustainable development’ have been primarily based on the three-pillar approach, which distinguishes between environmental, social, and economic dimensions of sustainable development [10]. The environmental dimension can be described as the sum of all biogeological processes and the elements involved in them. It demands preservation of the viability of ecological systems as the natural base sustaining human civilization [11]. The main obstacle regarding the concepts of sustainable development and sustainability is still their transformation and operationalization [9] at the practical level.

Sustainability criteria and their content should be linked to the understanding of what is sustainable development and sustainability. So far, sustainability criteria is not a clearly defined concept. It has been defined differently by different researchers. Simply put, sustainability criteria are requirements to the sustainable quality of a product and its sustainable production, which have to be fulfilled in order to acquire a sustainability status or certification [12]^a^. In a number of sources, the assessment function of sustainability criteria has been highlighted. Thus, Zink defines sustainability criteria as criteria that are applied to assess opportunities and risks deriving from economic, environmental, and social sustainability dimensions [13–15]. Koplin et al. point out that sustainability criteria of an environmental character form requirements placed on suppliers aiming to reduce the input of natural resources and minimize environmental risks by improving the efficiency of suppliers [16]. To my mind, Koplin's argument can be even expanded to other groups of the involved actors, e.g., to producers at different stages of the production process.

Goldschmidt et al. underline that sustainability criteria can be of a qualitative or quantitative nature [17,18]^b^. This research group also stresses that sustainability criteria are not static and often require continuous assessment and modification over time [17]. In some cases, sustainability criteria can set an upper limit to the use of natural resources and provide some institutional guidance [18]. Spangenberg highlights that sustainability criteria can be of much help in detecting unsustainable trends and effects, thus identifying unsustainable policy approaches, e.g., policies increasing resource use or societal disparities [11].

Sustainability criteria can be binding, when included in a legal framework. They can also be a part of a voluntary sustainability standard. Frameworks with sustainability criteria, primarily of a voluntary, non-obligatory character, which have been initiated by private actors, exist in different branches, such as biofuel, forestry, coffee, and cotton industries. Sustainability criteria are usually developed to serve certain purposes. These purposes have to be clarified, so that it will be easier to understand and interpret the content of the sustainability criteria. Most frameworks with sustainability criteria have a hierarchical structure, in which the main goal of the framework is transformed into a number of principles. Sustainability criteria are later formulated in such a way that they encourage and facilitate the fulfillment of the principles. The development of a legal framework or a sustainability standard with sustainability criteria should involve the setup of an organizational structure and an update of a minimum set of the criteria, as well as a system for accrediting the competence of private actors, who help to control the fulfillment of the criteria at different stages.

Barriers in the efficient function of sustainability criteria can occur because of different implementation approaches among the involved actors in different regions and development of weak overlapping legal frameworks and voluntary sustainability standards for the same product [19].

### Indicators for sustainability criteria

In a general sense, an indicator is a parameter which points to provide information about and describe the state of a phenomenon [9,20]^c^. An indicator should be clearly formulated and relatively simple to apply. Indicators for sustainability criteria are tools used to check and evaluate the fulfillment of sustainability criteria, as well as progress towards sustainability. Indicators can provide quantitative measurement and qualitative assessment of human activities and their impact on the surrounding world [21]. It should be observed that not all legal frameworks and sustainability standards use the concepts ‘sustainability criteria’ and ‘indicators’ in the same way. Sometimes, sustainability criteria are already measurable units, and indicators are missing [22].

Indicators that deal with the fulfillment of sustainability criteria should be linked to sustainability goals and objectives of the framework. They should reflect the performance of the sustainability criteria. The choice of indicators should be transparent. Working out well-functioning indicators takes time, and compromises have to be made. Often, this process requires intensive data collection, monitoring from multiple locations and repetition of these actions at appropriate time intervals.

There is not an alignment or consensus among the different organizations and scientists involved about the most appropriate or useful set of indicators. There are different interpretations, methods, and approaches for developing and using them. It can be recommended that indicators have the following characteristics:

1. Accurately reflective of the process or function they present;
2. Sufficiently sensitive to pick up changes over time and among different farming systems;
3. Feasible to measures in terms of time, expense, and level of skills required; and
4. Understandable and relevant for end users [21].

Many researchers differentiate between core and supplementary indicators for sustainable production [23]. Core indicators are an average set of indicators that can be applied at any company or facility. They are simple and easy to use, based on available data and commonly measured aspects of production [23]^d^. Supplementary indicators are an open set, and they vary between companies and facilities. These indicators introduce some flexibility by addressing additional and production-specific aspects [23]. From the environmental point of view, it is important that indicators, both core and supplementary, consider global environmental issues, such as global warming, acidification, water shortage, and biodiversity. The function of indicators can be improved, if they are designed to reflect performance in terms of a more complete set of system attributes, which includes measures of productivity, resource-use efficiency, and robustness [24].

A clear trend towards standardization of indicators within different branches is observed. Core indicators are not considered more important than supplementary indicators. They are seen as an initial step in searching for common measures for sustainable products and their sustainable production. Results from their practical use can show which indicators need to be modified and which function well for the majority of the involved actors [23].

A preliminary list of core indicators for sustainable products and their sustainable production can include the following parameters:

1. Energy and material use during the production process: energy and materials are conserved and the form of energy and materials applied are most appropriate for the desired result.
2. Natural environment, including human health: wastes and ecologically incompatible by-products are continuously reduced, eliminated, or recycled; chemical substances, physical agents, technologies, and work practices that present hazards to human health or the environment are also continuously reduced or eliminated.
3. Economic performance: management is committed to an open, participatory process of continuous evaluation and improvement, focused on the long-term economic performance.
4. Products: products and packaging are designed to be safe and ecologically sound throughout their life cycle; services are designed to be safe and ecologically sound [25].

Risks should not be transferred between different aspects of sustainable production, like between energy use and economic performance [26]. Possibly, not every producer that decides to comply with these indicators would apply them for each production stage [27]. Using indicators of sustainable production is a process of continuous improvement. It can be recommended to start using simple and easy to implement strategies of compliance and resource efficiency and later move towards more complex indicators, addressing environmental and social effects, supply chain, and lifecycle impacts [23].

There are different approaches, methods, and interpretations for developing and using indicators. Conclusions on the basis of indicator values are typically made answering the question of whether numerical values are higher or lower than before, and rarely in terms of whether the differences are likely to be functionally significant. As an example, measures of soil organic matter are the basis for most sustainability and soil quality assessments, being seen as an integrative indicator for soil properties, such as moisture-holding capacity, physical structure, and nutrient supply capacity. The numerical level that would be considered good, or what change in soil organic matter levels constitutes a significant functional change, has not been established [28].

Assessments of how sustainability criteria have been fulfilled, made with the help of one indicator, but applying different assessment methods and techniques, can vary substantially even when applied to the same criteria. An important question that still remains is how to make a holistic assessment of the relative sustainability of different systems given the multiple indicators that represent various sustainability goals and objectives. One practice is to combine individual indicators into an index, based on some additive procedures. A single index might obscure the values inherent in its calculation - which attributes weigh more than others - and can be particularly problematic, if the direction of change is positive for some measures and negative for some others [28]. An alternative is to evaluate the system performance without creating a single number, in which case, any tradeoffs or synergies might be identified [29].

The significance of constant reviews and assessments of sustainability goals, criteria, and indicators within a legal framework or a sustainability standard should be underlined. Reviews and assessments made with the help of different methods can lead to different results and interpretations, even when applied to the same framework or standard [29]. An assessment in a long-time perspective can add much to the efficient function of indicators. Some researchers stress that only through a regular review and revision of indicators and the whole system, within which they function, a continuous development can be achieved [30]. Improving the correlation between indicators, sustainability criteria, and sustainability goals is a priority for the future work [28].

As an example, programs on indicators for sustainable agriculture have been developed by many institutions, including the UN Food and Agriculture Organization, UN and World Bank, the International Institute of Sustainable Development, and the Land Stewardship Project. Experience from the USA shows that collaborative efforts among different groups of interested actors have been needed to develop an agreement about core indicators for measuring sustainability. The initiative with the title ‘Field to Market: the Keystone Alliance for Sustainable Agriculture and the Stewardship Index Initiative for Specialty Crops’ can serve as an illustration here [29].

### Control of the fulfillment of sustainability criteria in legal frameworks

Control of how sustainability criteria are fulfilled and its quality are very important. Thoroughly elaborated regulations on control mechanisms and their components, such as monitoring, reporting, verification, and transparency [31], should be included into legal frameworks and voluntary sustainability standards. The involved actors, e.g., in the case of transport biofuels, whose actions should be controlled in the first turn, are biofuel producers, suppliers, and distributors.

Westerlund differentiates between three main approaches to the environmental control: precautionary or best available technology (BAT approach), environmental quality approach, and combined approach [32]. According to him, the pure best available technology approach focuses on what people and their activities can manage economically, without having to abandon a project [32]. The environmental quality approach reflects the maximum of stress and change that the environment is supposed to manage. The most common strategy for this is to lay down environmental quality standards and critical load standards, for example, for water, air, and soil [33]. The combined approach presents a combination of the two above mentioned approaches [33].

The best available technology approach is typically an activity-oriented approach, and its requirements are related to what the activities can bear. The pure environmental quality approach is an environment-oriented approach. The requirements here are related, directly or indirectly, to what the environment can tolerate [34]. Westerlund means that it is very important whether we create requirements based upon what the activities can bear, or what the environment can bear. This issue reflects balancing between the actor-related and the reactor-related perspectives [35]. Among the advantages of the third, combined approach, there is a possibility to view pollution as illegal, when it is beyond the environmental quality standards [34].

Westerlund stresses that the methodology of environmental law is not restricted to constructing environmental legal frameworks. It is also about creating efficient control systems that include legally based means of control, ranging from soft and flexible instruments to hard conduct-regulating law [36]. Westerlund underlines that controlling humans is difficult, because ‘humans can act and many of them do so, in order to avoid or disobey control’ [37]. In one of his latest works, Westerlund uses the term ‘environmental control system’, when he discusses control of how legal requirements are fulfilled. He refers this term primarily to societal control, which is initiated and regulated by authorities, and to the parliament as the legislator [37]. In line with this, Westerlund distinguishes a basic structure for environmental control systems, which should include certain types of policy instruments, such as education, information, economic incentives, pollution fees, and conduct-related regulations [38]. Policy instruments may be soft and flexible, but they are normally not functional unless backed up with law [36], which highlights special capacities and functions of law.

According to the approach of Westerlund, certain structural elements in a legal framework are almost indispensible, in order to facilitate the implementation of its requirements. In the case of a framework with sustainability criteria that addresses sustainable production in different parts of the world, control of the fulfillment of sustainability criteria is an important issue to have regulations about. Other researchers highlight that the immediate focus for a policy with sustainability criteria should be on implementing the necessary controls and conditions that will enable the addressed industry to develop sustainably [39, 40].

My personal opinion is that a legal framework with sustainability criteria should be related to what its control mechanisms can achieve, in order for the whole system to remain functional. This point of view can be discussed on the basis of the EU example for transport biofuels that relies much on the commitment of actors outside the EU. The behavior of these actors is very difficult to control by the EU administrative bodies. This can be seen as a weakness of the EU approach.

One of the possible control mechanisms for the fulfillment of sustainability criteria is reporting obligations of the involved actors. This mechanism implies that regulations should be introduced in a legal text about what actors are obliged to make reports and what issues the reports should include. Practical information that explains these regulations can be placed in non-binding attachments to a legal act [41].

Within the EU framework for transport biofuels, the following groups of the involved actors can be required to make reports at the national level: biofuel producers, importers, deliverers, distributors, and users. Distributors seem to be the most appropriate group [42]. There are guidelines at the national level from the Swedish Energy Authority about what groups of actors are obliged to submit information about the use of sustainable biofuels to the national official energy statistics (see Swedish national documents STEMFS 2006:1 and STEMFS 2007:1). The list includes producers, importers, and distributors, though this list should be analyzed further. Information about to what extent the sustainability criteria are fulfilled is better collected at the beginning of the production chain. Information about the amount of sustainable biofuels delivered and used is better collected at the end of the production chain [42]. Reporting of the latter type will support calculations of the amount sustainable biofuels used by the EU member states [43].

An obligation to submit a report to the national authority may imply that the involved actor is to hand in information about how much biofuels that fulfill the EU sustainability criteria have been delivered and used. The actor has to prove that the declared amount of biofuels answers the requirements of the EU sustainability criteria. The obligations to submit the report should include that the handed-in information has been controlled by an independent auditor or inspector and that reporting has been made within the set time frames. The auditing can be done in parallel to the financial sector: the auditor should check all documents and inspect a sample of farmers and traders. In case of biofuels of an agricultural origin, it is important to check whether the production land for biofuels has indeed been farm land before and not a tropical forest of a high biodiversity value [44].

The system of control and reporting on the fulfillment of the sustainability criteria should be transparent. There should be opportunities for those who are outside the control mechanisms to be able to look over their function and to have access to the necessary information. The system of control should be reliable and trustworthy. Economically sensitive information can be protected with the help of security regulations [45].

A legal framework with sustainability criteria should contain regulations on which administrative bodies must be established for the enforcement of the sustainability criteria, as well as for the supervision and control of their fulfillment. Rights and obligations of these bodies should be legally defined [46]. As an example in Sweden, the Swedish Energy Authority can function as an administrative body that enforces, supervises, and controls the fulfillment of the EU sustainability criteria for transport biofuels at the national level. If it is needed, this administrative body can require cooperation from other authorities at the national level, such as the Swedish Agricultural Authority, the Swedish Nature Protection Authority, and the Swedish Forestry Authority [47].

As a practical example based on the EU approach to sustainable biofuels, a UK supplier of transport biofuels, who is using ethanol from Brazil, has to notify the quantities of sustainable biofuels to the UK authorities. To show that they are sustainable according to the EU regulations, the supplier can join a voluntary sustainability standard that has been accepted by the EU. The supplier has to make sure that throughout the production chain, all records for biofuels have been kept by the involved actors, such as the trader he or she buys the biofuels from, by the ethanol plant the trader buys the biofuels from, and by the farmer who supplies the ethanol plant with sugar cane. It can be recommended that this control is made before the supplier joins the voluntary sustainability standard with sustainability criteria and at least once a year after that.

Reasonable balance should be achieved between what is advisable for control mechanisms to function as they should and what the involved actors are able and capable of doing. The involved actors should be able and capable of implementing the legislated control mechanisms, as well as of fulfilling the requirements for monitoring, transparency, and reporting. Thus, the actors should be able to collect the required scientific data and to make calculations and assessments required in the report.

### Monitoring the fulfillment of sustainability criteria

In order to reduce uncertainties that surround the fulfillment of sustainability criteria, their influence on the market development, their impacts on sustainability, and their indirect effects on other markets and policies [48], continuous monitoring of how sustainability criteria are functioning is desirable. A voluntary sustainability standard might require an explicit confirmation of the host state that a project in its territory contributes to sustainable development [49]. It is advisable that monitoring of how the sustainability criteria are fulfilled is transparent [50], which implies that all necessary information is freely available and directly accessible to those who are affected by sustainability criteria and their implementation. The public should also have access to a sufficient amount of simplified information [51]. As an example, this can be achieved by making all relevant documentation available and easily accessed on the Internet. In the sector of transport biofuels, the name, size, and location of plantations, where crops for transport biofuels are farmed, can be made public. A database can be created for monitoring how crops for transport biofuels and biomass, produced on their basis, are used [52].

Monitoring should take place at all stages of the production chain, though this is difficult to achieve. In the case of transport biofuels, monitoring should go further than only monitoring of the raw material production. It should also include monitoring of the supply and distribution chains. To make monitoring more efficient, the reliability of data sources may be asserted by some groups and challenged by others [53]. Before the implementation of an extensive monitoring system, it should be considered whether it is worth the effort. Obligatory reporting can be seen as a simple and efficient tool for monitoring [54]. It has the potential to collect a broad spectrum of precise data [52]. Requirements to prove the correctness of the collected material can be stated. In-company checks and field visits can also be a part of the monitoring process.

Understanding that monitoring is an important structural element in the implementation of sustainability criteria, it can be anticipated that legal frameworks and voluntary sustainability standards with sustainability criteria include well-functioning monitoring mechanisms. The quality of monitoring should be regularly revised and improved.

### Costs of following sustainability criteria

Introduction of sustainability criteria should not be too expensive for those who follow them [55]. The costs of the involved actors typically involve costs of the implementation of the sustainability requirements expressed through the sustainability criteria and of local control of their fulfillment, such as costs of independent auditing. There are also costs of getting certified according to one or several voluntary sustainability standards that might be compatible with the existing legal frameworks for a product. These are direct costs. Indirect costs can be related to changes in production and management practices [56], though this division is rather theoretical. The existing dispersion of indirect costs can be explained by the willingness to apply sustainability criteria and different ambition levels [57].

The costs of getting certified according to a voluntary sustainability standard are generally not high, because certifications should be open to a broad spectrum of interested actors. In spite of this, critics often find fault with these costs, meaning that they are high [55,58]. These costs can form a serious obstacle to small-scale producers and producers in developing countries. A possible practical solution can be to allow group certifications, in which certification costs can be shared by the participants [59]. Another approach is to develop sustainability criteria that are effective in relation to their purpose, easy to follow, and cheap. In any case, a balance between the efficiency and coverage of the sustainability criteria, and the costs that their users are interested to pay should be achieved [57]. Both direct and indirect costs have a limited influence on the society, because it is the user of a voluntary sustainability standard who is aimed to pay [60].

Regarding the costs of following legally binding sustainability criteria, it can be suggested that these costs correspond to the common patterns of the establishment and implementation of a legal framework. This would include costs of creating a legal framework with sustainability criteria; costs of building up an administrative apparatus to enforce, supervise, and control the fulfillment of the sustainability criteria; costs of the evaluation of the achieved results and reconsideration; as well as costs of sanctions, if the sustainability criteria have not been fulfilled as they should. A large amount of costs, as in the case with voluntary sustainability standards, would be laid on the producers, distributors, and suppliers of the sustainable product. These groups of the actors should bear the costs of fulfilling the sustainability criteria and of proving compliance with them.

Taking the EU legal framework for transport biofuels as an example, its costs for the society include in the first place costs of the EU and national governments to create and maintain efficiently functioning legal frameworks with sustainability criteria, as well as costs of subsidies that the EU grants [61]. Following the EU sustainability criteria is expected to increase the costs of biofuel production [61], partially because certain administrative and technical barriers might need to be overcome. Contradictions with the WTO regulations and uncertainty about independent auditing as a control mechanism are examples of administrative barriers for the implementation of the EU sustainability criteria that would require additional costs.

### Groups of actors responsible for setting and supporting sustainability criteria

Different groups of actors can be distinguished, who can be responsible for the creation of sustainability criteria and support of their implementation. Among them there are (1) international institutions, (2) states and their governments, (3) independent bodies established by states, (4) NGOs, (5) producers, (6) users, and (7) research institutions. Potential contributions of these groups are explained below.

1. International institutions
  International institutions possess wide possibilities to establish and support sustainability criteria for different industries.
2. States and their governments
  There is an opinion that national governments should support sustainability initiatives and the establishment of sustainability criteria. Environmental costs of a product should be internalized in market prices. NGOs should be involved in monitoring activities. Minimum prices should be used to guarantee a fair income and care for the environment [62].
  Calls to combine efforts from international and national institutions have been made more and more explicit. Hopes have been expressed that this approach has the potential to neutralize negative effects of global free trade and rein in corporate power [63]. As an example, the Clean Development Mechanism (CDM) that is an international initiative requires an explicit confirmation of the state, which hosts a project, that its development will contribute to the sustainability of the state's territory. An approval of the host state cannot, however, replace the agreed sustainability criteria. Risks of increased bureaucracy and decision-making opportunities for developing countries with weakened governance need be assessed [64].
3. Independent bodies established by states
  It should be encouraged that independent organizations established by states share their opinions during negotiations on sustainability criteria and later on check to what extent the criteria have been fulfilled. A supposition has been made that already functioning legal frameworks and voluntary sustainability standards will be interested in getting support from independent organizations established by states, for example, for carrying out certification and control activities [65].
4. NGOs
  It has been observed that the role of NGOs, which missions often coincide with the tenets of sustainable products in establishing sustainability standards and eco-labels, is very high. Warnings should be made against putting much reliance on NGOs only in setting up and supporting sustainability criteria, because NGOs might lack professional knowledge, resources, and contacts with the industry [62, 66].
5. Producers
  Producing companies may refuse to be transparent about the amount and quality of sustainable products they are supporting, as well as about their production methods [67]. In these cases, interests of the market and consumers in having clear and concise access to sustainable products and information about them may prevail [68].
6. Users
  An opinion has been expressed that it is the product user who has to prove that the consumed product is sustainable, and the user is the primary ‘problem owner’ [65]. This position is very questionable, because possibilities of an ordinary user to assess the sustainability of a product or its production methods can be very limited.
7. Research institutions
  Contribution of research institutions and scientists is very important, because they provide the necessary scientific ground to the content of sustainability criteria and assessment of their fulfillment.

The importance for different groups of actors to collaborate, as well as transparency and consumers' access to information about the fulfillment of the sustainability criteria, should be highlighted. More research on these issues is needed.

### An example of making a framework with sustainability criteria more efficient

In this section, a list is provided, which includes factors that facilitate the implementation and use of sustainability criteria through the participation of different groups of the involved actors. The role and possible impacts of each factor are not obvious and should be analyzed further. The list has initially been made for bioenergy, but it can be generalized and used for other products, where the achievement of sustainability criteria is necessary.

1. Participation of the industry, especially large-scale producers, and international standardization and accreditation organizations;
2. NGOs should be involved in the negotiation process and monitoring activities, though their lack of professionalism should be thought through;
3. Low barriers of entry for small-scale producers and producers from developing countries, including costs and knowledge, enhance their participation;
4. Monitoring should be performed by professional and commercial organizations;
5. Monitoring should include regular field inspections and not based on paper checks only;
6. The competition and transparency within the co-existing legal frameworks and voluntary sustainability standards, and the content of their sustainability criteria should be supervised;
7. Gradual standardization and certification procedures improve the involvement of industry;
8. Linking governmental subsidies and price premiums to the fulfillment of sustainability criteria safeguards financial incentives;
9. Anonymous trade should be diminished. This will improve the terms of trade for producers and create transparency for consumers and other interested actors; and
10. Participation of the industry and raising consumer awareness should be increased.

### Co-operation between the groups of the involved actors

A perfect model that governs how to create, enforce, and implement legal frameworks and voluntary sustainability standards with sustainability criteria does not seem to exist. Sustainability policies and projects are still not widely used or, regarding some products, can be absent [62]. A suggestion has been made to encourage producers to consult with governments, research institutes, and NGOs [69], with the purpose to arrive to a shared solution through collaboration. Standard setting processes can be conceptualized as new forms of contract where a state, rather than being directly involved between the parties, provides a form of basic guarantee, while NGOs and companies are in charge of making up the agreements [70].

Voluntary sustainability standards are usually initiated and developed by large-scale producers and NGOs. The experience in the coffee sector can serve as an example here. The advantage of this approach is that the non-obligatory involvement of these groups of the actors does not limit trade.

Members of the bodies that answer for the enforcement and fulfillment of sustainability criteria can suggestively include NGOs, producers, scientists, and consumers. Obligations of these management bodies can embrace the establishment of sustainability criteria, assessment of their fulfillment and updating, development and improvement of indicators, and control of environmental sustainability and claims, as well as promotion, harmonization, and accreditation of the co-existing sustainability standards [71]. Certain independence of the management bodies in their decision-making is desirable.

## Conclusions

The more we research the notion of sustainability criteria and critical issues, which their practical implementation and use involve, the better our efforts can be to improve them. It is very possible that there is no perfect practical solution for using sustainability criteria in legal frameworks and voluntary sustainability standards, which usually co-exist and may overlap.

In the article, the existing definitions of sustainability criteria have been explored, the notion of indicators for sustainability criteria has been investigated, and the issue of costs for following sustainability criteria has been researched. It has been discussed what groups of actors can be responsible for setting and supporting sustainability criteria. The main focus has been put on the function of sustainability criteria as a tool to promote sustainable products and their sustainable production. Knowing that these issues can be researched from the perspective of different disciplines, the perspective of law and incorporation of sustainability criteria in legal constructions have been chosen. Several important reflections and conclusions have been made.

It can be presupposed that voluntary sustainability standards and similar measures, like eco-labels, should play an important role in addressing the issues that obligatory sustainability criteria are not able to deal with efficiently. However, non-obligatory means to promote and safeguard sustainability have their own limitations. Voluntary sustainability standards can lack efficiency to influence circumstances and developments that take place on a level higher than a company level. For example, not many voluntary sustainability standards can do without setting criteria for the production effects on climate, though these effects can hardly be satisfactorily measured at the level of an average producer.

In order for a legal framework or a voluntary sustainability standard with sustainability criteria to function as it has been aimed for, the content of the sustainability criteria should fulfill certain requirements. Thus, the criteria should be understandable. It should be possible to implement and supervise them, as well as to have control of their fulfillment. The involved actors should be willful to follow the criteria and the whole policy package, to which the criteria refer.

The efficiency of a legal framework or a voluntary sustainability standard with sustainability criteria cannot be assessed according to the number of the sustainability criteria included. ‘The more, the better’ seems to be an incorrect approach here [53]^e^. A clear difference should be made between theoretically elaborated lists of sustainability criteria, which ought to exist, and sustainability criteria aimed for practical implementation. The latter group should be less extensive and complicated.

The research has shown that the use of sustainability criteria has a number of typical advantages. For example, they assure sustainability in the long perspective and secure investments. Sustainability criteria is not a clearly defined concept. They have been defined differently by different researchers. It can be recommended to work out a homogeneous definition. The content of sustainability criteria should be linked to the understanding of what sustainable development and sustainability in each particular branch are. Sustainability criteria are usually developed to serve certain purposes. These purposes have to be explained, so that it is easier to interpret and fulfill the sustainability criteria. In some cases, sustainability criteria can set an upper limit to the use of natural resources and provide institutional guidance. It is desirable that sustainability criteria are applied at initial stages of an industry development.

Sustainability criteria should have clear delimitations, which can be supported by clarifying indicators. Indicators for sustainability criteria are tools used to check and evaluate the fulfillment of sustainability criteria, as well as progress towards sustainability. Among the recommendations for using indicators for sustainability criteria, it can be suggested that indicators are linked to sustainability goals and objectives of the framework with sustainability criteria. They should reflect the performance of the sustainability criteria. The choice of indicators should be transparent.

Control of how sustainability criteria are fulfilled and its quality are very important. Thoroughly elaborated regulations on control mechanisms and their components, such as monitoring, reporting, verification, and transparency, should be included into legal frameworks and voluntary sustainability standards. One of the possible control mechanisms for the fulfillment of sustainability criteria is reporting obligations of the involved actors. Independent auditing can be done in parallel: an auditor can check all necessary documents and inspect a sample of farmers and traders. A framework with sustainability criteria should be related to what its control mechanisms can achieve, in order for the whole system to remain functional.

The system of control of the fulfillment of the sustainability criteria should be reliable, trustworthy, and transparent. There should be opportunities for those who are outside the control mechanisms to be able to look over their function and to have access to the necessary information. To have more order, a legal framework with sustainability criteria should contain regulations on which administrative bodies must be established for the enforcement of the sustainability criteria, as well as for the supervision and control of their fulfillment.

In order to reduce uncertainties that surround the fulfillment of sustainability criteria, their influence on the market development, and their impacts on sustainability, continuous monitoring of how sustainability criteria are functioning is desirable. Monitoring should be transparent. It should take place at all stages of the production chain, though this is difficult to achieve.

Introduction of sustainability criteria should not be too expensive for those who follow them. Costs of getting certified according to a voluntary sustainability standard are generally not high, because certifications should be open to a broad spectrum of interested actors. A large amount of costs is laid on the producers, distributors, and suppliers of the sustainable product.

Different groups of actors at different levels can be responsible for setting and supporting the implementation of sustainability criteria. Among them are international institutions, states, independent bodies established by states, NGOs, producers, and users. Co-operation between these groups is very important, aiming to arrive to shared and more efficient solutions. A governance model can be suggested, when a state, instead of being directly involved between the parties, provides a form of basic guarantee, while NGOs, producers, and users are in charge of making up agreements. Warnings should be made against relying too much on NGOs in setting up and supporting sustainability criteria, because NGOs might lack professional knowledge, resources, and required contacts with producers.

It is important to consider whose interests are primarily taken into account, when a list of sustainability criteria is worked out. The content of the list would differ, depending on whether its dominating purpose is to protect the environment, or industrial development, or whether the interests of the consumers are prioritized. For the future, it can be suggested that more comprehensive, practically applicable frameworks and standards with sustainability criteria in different branches need be developed, tested, and researched.

## Method

The research method applied in this article can be called an analytical method in the qualitative research approach. It was focused on the data collection and its subsequent analysis. The process was not linear or straightforward. It had an iterative character, going back and forth. The ways to generate new knowledge included the analysis of the sources that contained research of the nature of sustainability criteria and special features of their use. Where necessary, the collection and analysis of the relevant non-binding policy documents, scientific reports, and the traditional legal sources, such as legal frameworks and preparatory legal acts for them, were made. Elements of the indicative analysis were added, which involved reasoning from smaller case-specific conclusions to general principles and larger theoretical systematizations. This generated rich, detailed, and comprehensive research results that can be applied to sustainability criteria for different products. The design of the article and presentation of the research results suited the requirements of the present scientific journal.

## Endnotes

^a^An initial research on this topic can be found in [12].

^b^The same opinion has been expressed in [18].

^c^The literature does not always distinguish between the terms sustainability criteria and their indicators. In some cases, these terms have been used interchangeably.

^d^For example, water use, energy use, employee job satisfaction, and company donations.

^e^This can be compared with the conclusions of [11].
